# Designing of interferon-gamma inducing MHC class-II binders

**DOI:** 10.1186/1745-6150-8-30

**Published:** 2013-12-05

**Authors:** Sandeep Kumar Dhanda, Pooja Vir, Gajendra PS Raghava

**Affiliations:** 1 Bioinformatics Centre, CSIR-Institute of Microbial Technology, Sector 39A, Chandigarh 160036, India

## Abstract

**Background:**

The generation of interferon-gamma (IFN-γ) by MHC class II activated CD4^+^ T helper cells play a substantial contribution in the control of infections such as caused by *Mycobacterium tuberculosis*. In the past, numerous methods have been developed for predicting MHC class II binders that can activate T-helper cells. Best of author’s knowledge, no method has been developed so far that can predict the type of cytokine will be secreted by these MHC Class II binders or T-helper epitopes. In this study, an attempt has been made to predict the IFN-γ inducing peptides. The main dataset used in this study contains 3705 IFN-γ inducing and 6728 non-IFN-γ inducing MHC class II binders. Another dataset called IFNgOnly contains 4483 IFN-γ inducing epitopes and 2160 epitopes that induce other cytokine except IFN-γ. In addition we have alternate dataset that contains IFN-γ inducing and equal number of random peptides.

**Results:**

It was observed that the peptide length, positional conservation of residues and amino acid composition affects IFN-γ inducing capabilities of these peptides. We identified the motifs in IFN-γ inducing binders/peptides using MERCI software. Our analysis indicates that IFN-γ inducing and non-inducing peptides can be discriminated using above features. We developed models for predicting IFN-γ inducing peptides using various approaches like machine learning technique, motifs-based search, and hybrid approach. Our best model based on the hybrid approach achieved maximum prediction accuracy of 82.10% with MCC of 0.62 on main dataset. We also developed hybrid model on IFNgOnly dataset and achieved maximum accuracy of 81.39% with 0.57 MCC.

**Conclusion:**

Based on this study, we have developed a webserver for predicting i) IFN-γ inducing peptides, ii) virtual screening of peptide libraries and iii) identification of IFN-γ inducing regions in antigen (http://crdd.osdd.net/raghava/ifnepitope/).

**Reviewers:**

This article was reviewed by Prof Kurt Blaser, Prof Laurence Eisenlohr and Dr Manabu Sugai.

## Background

The present vaccination strategies are contemplating subunit vaccine as an alternative to traditional attenuation approach. These subunit vaccines consist of a part of the pathogen to be used as vaccine, which generally include the peptides or proteins [1,2]. This novel strategy of vaccination has motivated the research towards development of subunit vaccines to combat a number of diseases like tuberculosis, malaria, anthrax, cancer and swine fever [3-7]. The major challenge in designing subunit vaccine is identification of antigenic regions (peptides or proteins) in the pathogen proteome that can induce desired immune response in the host organism, mainly human. Ideally one should experimentally check immune response for each possible fragment/peptide of pathogen proteome. In practice, it is not possible due to two reasons i) possible fragments are in the range of millions and ii) experimental techniques are costly and time consuming [8-11]. There is a need to assist experimental scientist using alternate approaches like computational techniques.

There is a tremendous change in the field of immunology in last few years due to exponential growth of new field immunoinformatics or computational immunology. In the last decade, numerous software, databases and web servers have been developed to identify antigenic regions that can activate various arms of the immune system like humoral, cellular and innate immunity. Broadly these *in silico* tools can be divided in following categories; i) linear/conformational B-cell epitopes for activating humoral response, ii) MHC class I/II binders, TAP binders, protease cleavage for understanding cell mediated immunity and iii) pathogen associated molecular patterns for activating innate immunity [12-40].

**Figure 1:**
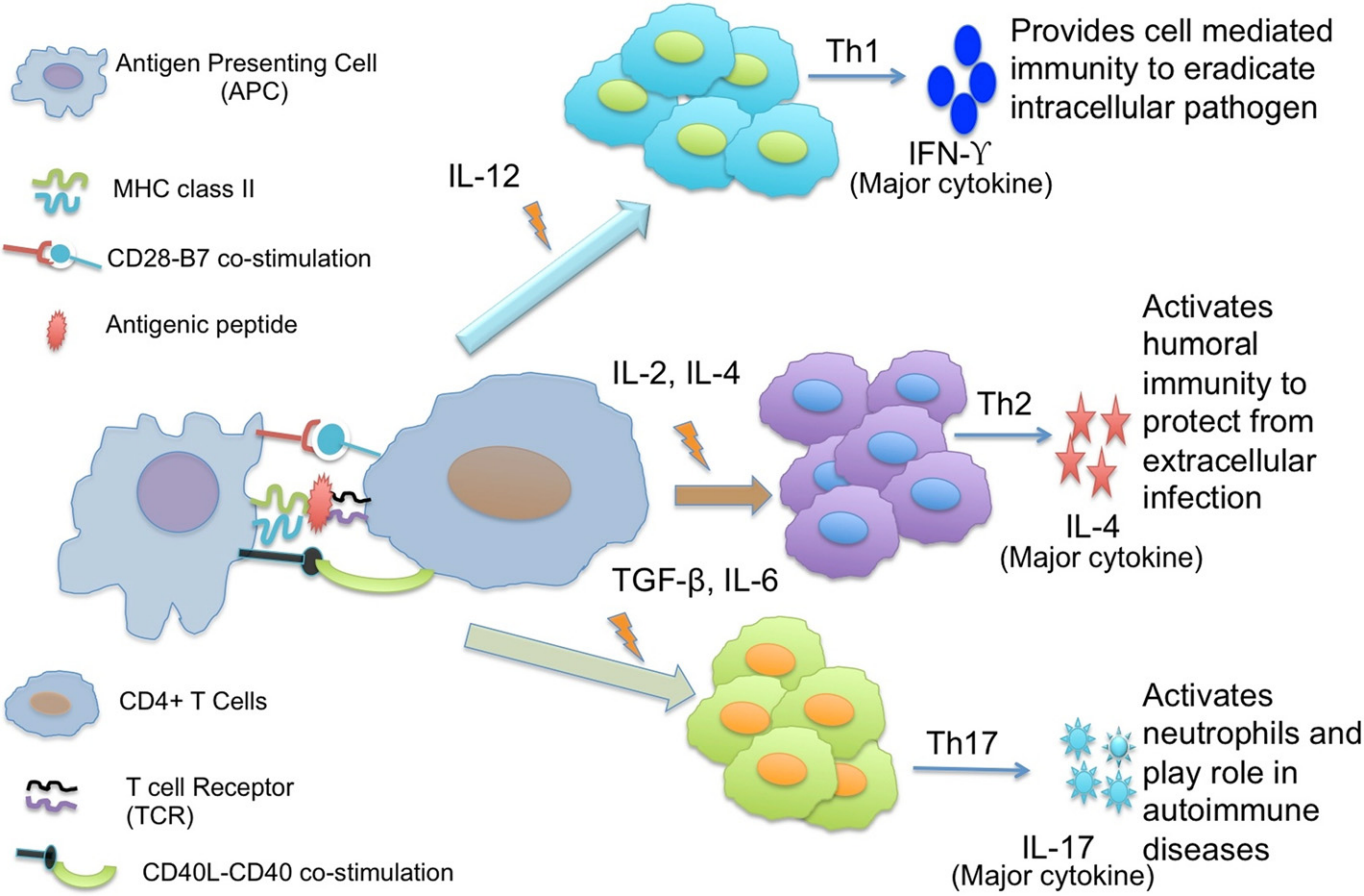
The schematic representation of CD4+ T cell differentiation into three principal subsets.

Identification of antigenic regions that bind MHC class II and activate T-helper cells are crucial for designing subunit vaccine. As activated T-helper cells release cytokines that activate cytotoxic T-cell and B-cells. There are different types of T-helper cells (e.g., Th1, Th2, Th17, iTregs) and each type of helper cell secrete specific type of cytokine [41-44] (Figure 1). For example, Th1 cells release IFN-γ and activates macrophages that are required to eradicate the intracellular pathogen like *Mycobacterium tuberculosis*[45-48]. T cells, NK cells, and NKT cells are the primary producers of IFN-γ, and it helps in fighting against bacterial, viral and tumor growth by regulating immune system. In order to design subunit vaccine or immunotherapy, one need to identify MHC class II binders that can activate IFN-γ inducing T-helper cells.

In past numerous methods have been developed to predict MHC class II binders that can activate T-helper cells. Best of author’s knowledge no method has been developed so far that can predict the type of T-helper cells will be activated, or type of cytokine will be released. The role of epitopes in deciding the immune response is well documented in literature [49-52]. In order to design subunit vaccine with more precision, there is a need to develop a method that can predict peptides that can activate specific type of cytokine. In this study, first time a systematic attempt has been made to predict IFN-γ inducing MHC class II binders or peptides.

## Methods

### Datasets

#### Main dataset

We extracted 10,433 experimentally validated MHC class II binders or T-helper epitopes from Immune Epitope Database (IEDB) [53]. Out of these 10,433 MHC class II binders, 3705 induced IFN-γ, whereas remaining 6728 unique peptides have not induced IFN-γ. Thus, our dataset contains 3705 positive examples or IFN-γ inducing peptides and 6728 negative examples or IFN-γ non-inducing peptides.

#### IFNgOnly dataset

This dataset has been created to resolve the issue, if a peptide is not inducing interferon-gamma, would it induce other cytokine after binding with MHC class II? The dataset was compiled from IEDB; we obtained 4483 MHC II binders or epitope that induce IFN-gamma only and 2160 epitopes which induce cytokines other than interferon-gamma. The numbers of IFN-γ inducing epitopes are greater in this dataset than our main dataset due to updation of IEDB in the mean time. While creating this dataset, we have removed the redundant and the epitopes which have induced two or more cytokines.

#### IFNrandom or alternate dataset

This is alternative dataset, where IFN-gamma inducing epitope were taken positive examples and equal numbers of peptides (3705) with same length variation from swissprot were generated in random fashion for negative examples. The model developed on this dataset would be very useful in discriminating the IFN-gamma inducing epitopes from the peptides for which MHC binding status is not known.

### Analysis of length and positional conservation of peptides

In order to understand the preference of length in positive and negative peptides, we used R-package for creating boxplot [54]. To understand position specific preference of each residue, we used two-sample logo software, where we created a two-sample logo from first 15 amino acids of N-terminal of complete peptides [55]. In this case, we removed all the peptides shorter than 15 residue length and remaining 89% peptides contained 2965 and 6336 peptides of positive and negative instances, respectively. On the other hand, in IFNgOnly dataset, there were 3682 epitopes in positive examples and 1641 epitopes remained in negative examples after applying the above filter.

### Motif based approach

Identification of functional motifs in peptides or proteins is extremely valuable in the field for functional annotation of proteins/peptides [56]. In this study, we used a powerful software called MERCI for searching exclusive motifs in positive and negative examples [57]. Although, MERCI uses positive and negative examples simultaneously as an input but at a time it gives motifs for the positive examples only. Therefore, we applied two-step strategy, where first we used IFN-γ inducing peptides dataset as positive and non-IFN-γ inducing peptide dataset as negative input and extracted motifs for IFN-γ inducing examples. Consequently, in order to extract motifs for the non-IFN-γ inducing examples, we used IFN-γ inducing examples as negative and IFN-γ non-inducing examples as positive input. In this way, we extracted motifs for both IFN-γ inducing and IFN-γ non-inducing examples. We have searched 100 degenerate motifs from the following three kinds of classification: i) None, ii) Koolman-Rohm and iii) Betts-Russell. The Betts-Russell classification could be further divided in to 3 categories: i) Polar, ii) Hydrophobic and iii) Small. These different classification methods produce different motifs in the both positive and negative peptides. Thus, we selected unique motif-containing peptide from both datasets, in order to calculate overall motif coverage in the dataset. The peptides of IFN-γ inducing and IFN-γ non-inducing examples containing positive and negative motifs were assigned as true positives and true negatives respectively.

### Amino acid compositions

In-house Perl scripts were used to calculate the amino acid composition, which encapsulate the intact epitope information in a fixed vector length as required by machine learning algorithm. The amino acid composition (MPC) creates a vector of 20 properties for each epitope using the following formula:

$$\begin{array}{l} \text{Compostition}\;\mathrm{of}\;\text{amino}\;\text{acid}\left(i\right)\\ \;=\frac{\text{Total}\;\text{number}\;\mathrm{of}\;\text{amino}\;\text{acid}\;\left(i\right)\times \;100}{\text{Total}\;\text{number}\;\mathrm{of}\;\text{all}\;\text{amino}\;\text{acid}\;\mathrm{in}\;\text{epitope}}\\ \;\text{Where}\;i\;\mathrm{can}\;\mathrm{be}\;\text{any}\;\text{amino}\;\text{acid} \end{array}$$

Similarly, di-peptide composition (DPC) resulted in a vector of 400 and was computed using the formula:

$$\begin{array}{l} \text{Compostition}\;\mathrm{of}\;\text{dipeptide}\left(i+1\right)\\ \;=\frac{\text{Total}\;\text{number}\;\mathrm{of}\;\text{dipeptide}\left(i+1\right)\times 100}{\text{Total}\;\text{number}\;\mathrm{of}\;\text{all}\;\text{possible}\;\text{dipeptides}\;\mathrm{in}\;\text{epitope}}\\ \text{Where}\;i\;\mathrm{can}\;\mathrm{be}\;\text{any}\;\text{amino}\;\mathrm{acid}\;\mathrm{and}\left(i+1\right)\;\mathrm{is}\;\text{dipeptide}\;\text{pair}\;\text{with}\;\text{next}\;\text{residue}\;\mathrm{in}\;\text{peptide} \end{array}$$

### Binary approach

We applied binary approach, in which positive and negative examples were converted into the binary patterns. Each amino acid represented by an unique vector of 20 dimensions (e.g. Ala by 1,0,0,0,0,0,0,0,0,0,0,0,0,0,0,0,0,0,0,0; Cys by 0,1,0,0,0,0,0,0,0,0,0,0,0,0,0,0,0,0,0,0) for different 20 standard amino acids. For example,15-residue long peptide represented by the 300 (15 X 20) dimensions of a vector as an input.

### Machine learning approach

In this study, SVM (Support Vector Machine) was applied for machine learning approach [58]. Based on the features (amino acid composition and length) generated above, the support vector machine was optimized at different parameters of various kernels (linear, sigmoidal and radial basis function), and the best-optimized model was selected for software implementation.

### Hybrid approach

In the hybrid approach, we combined the predictions from motif approach and machine learning approach. First of all, the sequences were separated that could be correctly predicted via motif based approach and the remaining sequences were then predicted using SVM. Various hybrid models were developed based on the type of vector inputs used for SVM-based prediction. Finally, the performance was evaluated by adding the truly predicted peptides from the motif-based method with SVM based predictions.

### Cross validation

To test the vigor of the model, it was evaluated with five fold cross validation, where the complete dataset was divided into five equal parts and out of these four parts were used for training and the remaining fifth part was used for testing. This process was repeated five times in such a way that each part was once used for testing and four times it was a part of training. The overall performances were calculated by averaging the result of each test. The best model was also validated on 10 fold cross validation. In cross validation for hybrid approach, the results of motifs were directly added in the five or ten fold cross validation through SVM based approach.

### Evaluation parameters

The performance of the model was evaluated in terms of sensitivity, specificity, accuracy and MCC^16^. These parameters were derived from the equations:

$$\begin{array}{l} \text{Sensitivity}=\frac{\mathrm{TP}}{\mathrm{TP}+\mathrm{FN}}\times 100\\ \text{Specificity}=\frac{\mathrm{TN}}{\mathrm{TN}+\mathrm{FP}}\times 100\\ \text{Accuracy}=\frac{\mathrm{TP}+\mathrm{TN}}{\mathrm{TP}+\mathrm{FN}+\mathrm{TN}+\mathrm{FP}}\times 100\\ \mathrm{MCC}=\frac{\mathrm{TP}\times \mathrm{TN}‒\mathrm{FN}\times \mathrm{FP}}{\sqrt{\left(\mathrm{TP}+\mathrm{FN}\right)\left(\mathrm{TP}+\mathrm{FP}\right)\left(\mathrm{TN}+\mathrm{FP}\right)\left(\mathrm{TN}+\mathrm{FN}\right)}} \end{array}$$

TP = True Positive, FP = False Positive, TN = True Negative, FN = False Negative.

## Results

### Examination of dataset

The peptides in the main dataset were obtained from 17,752 assays, where 5962 assays had shown to be positive for interferon-gamma secretion. These peptides were derived from 281 source organisms and were presented through 153 MHC alleles from 181 different host species/strains. On the other hand, the epitopes in IFNgOnly dataset were extracted from 15,778 assays. Out of these 15,778 assays 7302 assays have induced IFN-gamma and remaining 8476 assays have induced the secretion of other cytokine except interferon-gamma. The epitopes in IFNgOnly dataset were extracted from 394 different sources and presented through 183 MHC alleles in 232 host strains. The detailed analysis of epitopes with respect to MHC alleles, host strain and source organisms is available in supplementary excel sheet (Additional file 1).

### Data analysis

**Figure 2:**
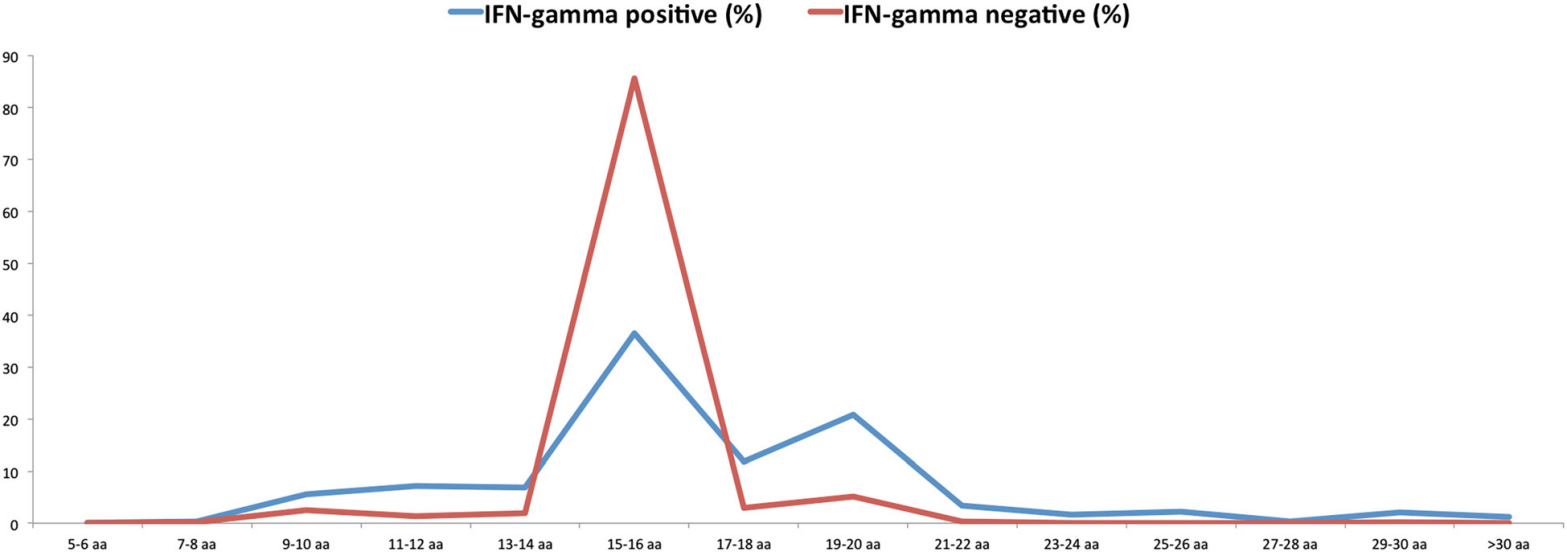
Bar graph showing length of peptide and their frequency in our main dataset.

**Figure 3:**
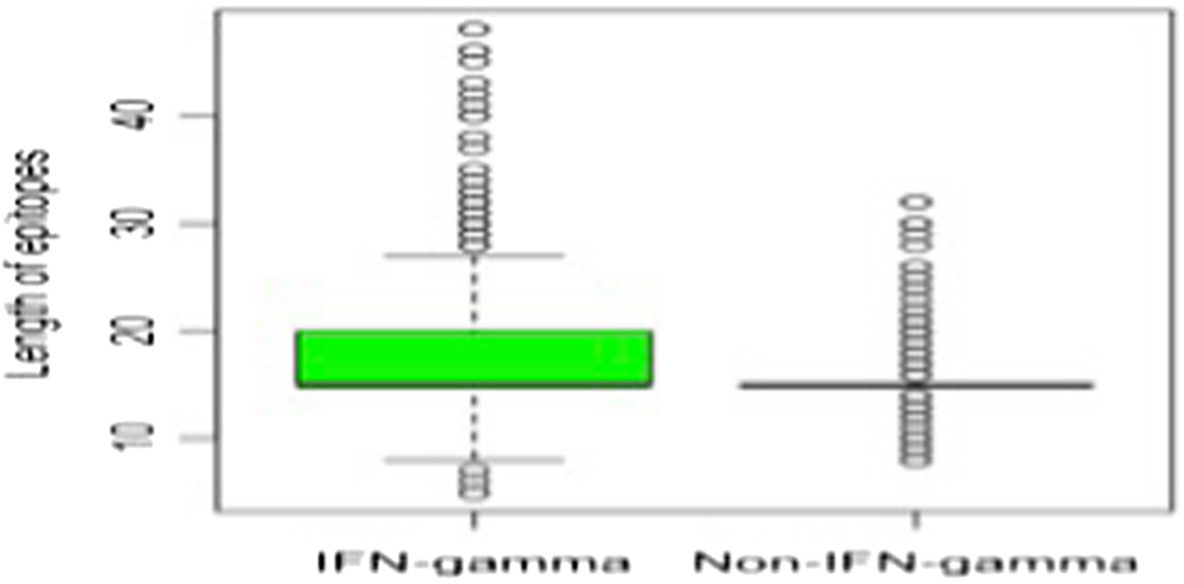
**Boxplot to showing the length-wise distribution of both type of MHC II binders (IFN-γ inducing and non-inducing peptides) in main dataset.** The dots are representing the outliers; the dotted line represents to cover the data and strong line displays median.

**Figure 4:**
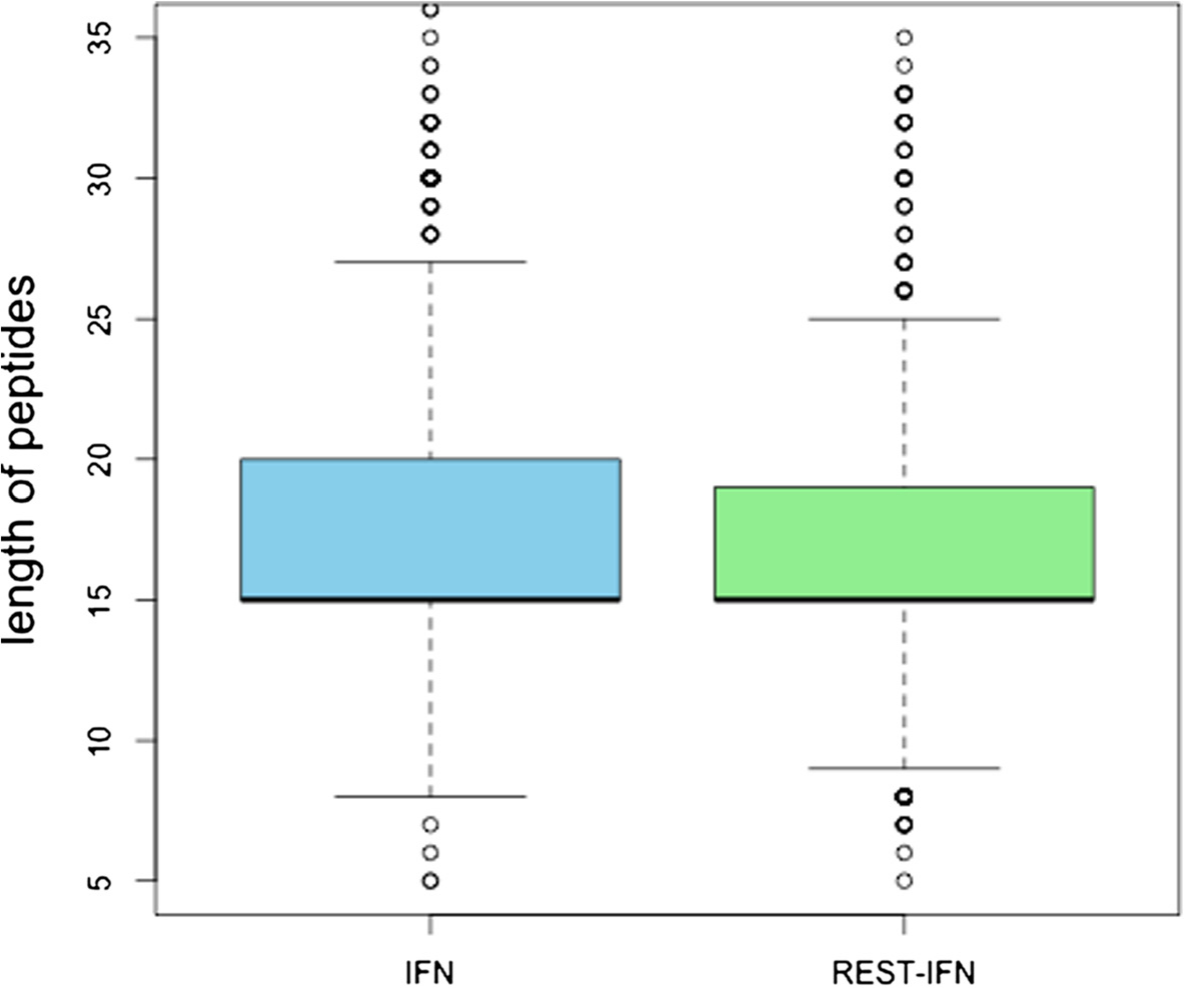
**Boxplot to represent the distribution of epitopes in IFNgOnly dataset.** Here dots are outliers. Blue box is having IFN-γ epitopes while green box comprises of the epitopes secreting rest of cytokine (except IFN-γ).

We analyze IFN-γ inducing and non-inducing peptides in main dataset to fish out the important features. It was observed that the length of peptides plays a prominent role in discriminating the IFN-γ inducing and non-inducing peptides (Figure 2). As shown in Figure 2, majority of the negative peptides fall within the range of 15–16 amino acids while most of the positive peptide have wide distribution from 13 to 22 residues. It can be deciphered from the boxplot (Figure 3) that the IFN-γ inducing and non-inducing peptides prefer different lengths. The whiskers of the boxplot denote the range of distribution that varies from 8 to 27 residues length in positive dataset while negative data clumped only at the residues length of 15. The green colored area in the box could be inferred as the skewness of the positive dataset toward the length more than 15 amino acid residues which means IFN-γ inducing dataset has significant peptides with length more than 15 amino acid residues. No data skewness was observed in IFN-γ non-inducing samples. We did not find any difference in length of peptides inducing IFN-γ from the peptides that have induced any other cytokine than IFN-γ present in our IFNgOnly dataset (Figure 4).

### Composition analysis

**Figure 5:**
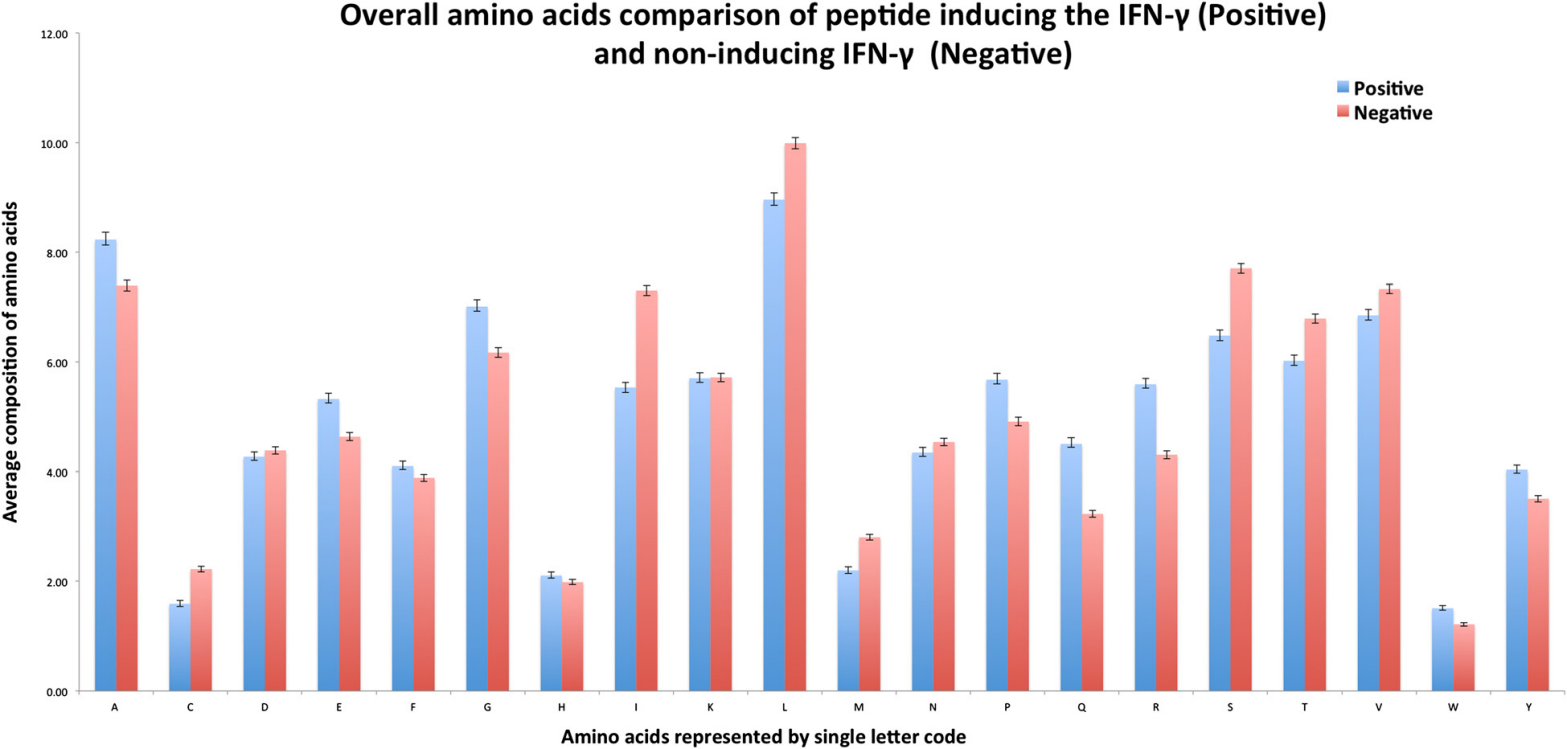
Amino acid composition of both class of MHC class II binders in main dataset.

**Figure 6:**
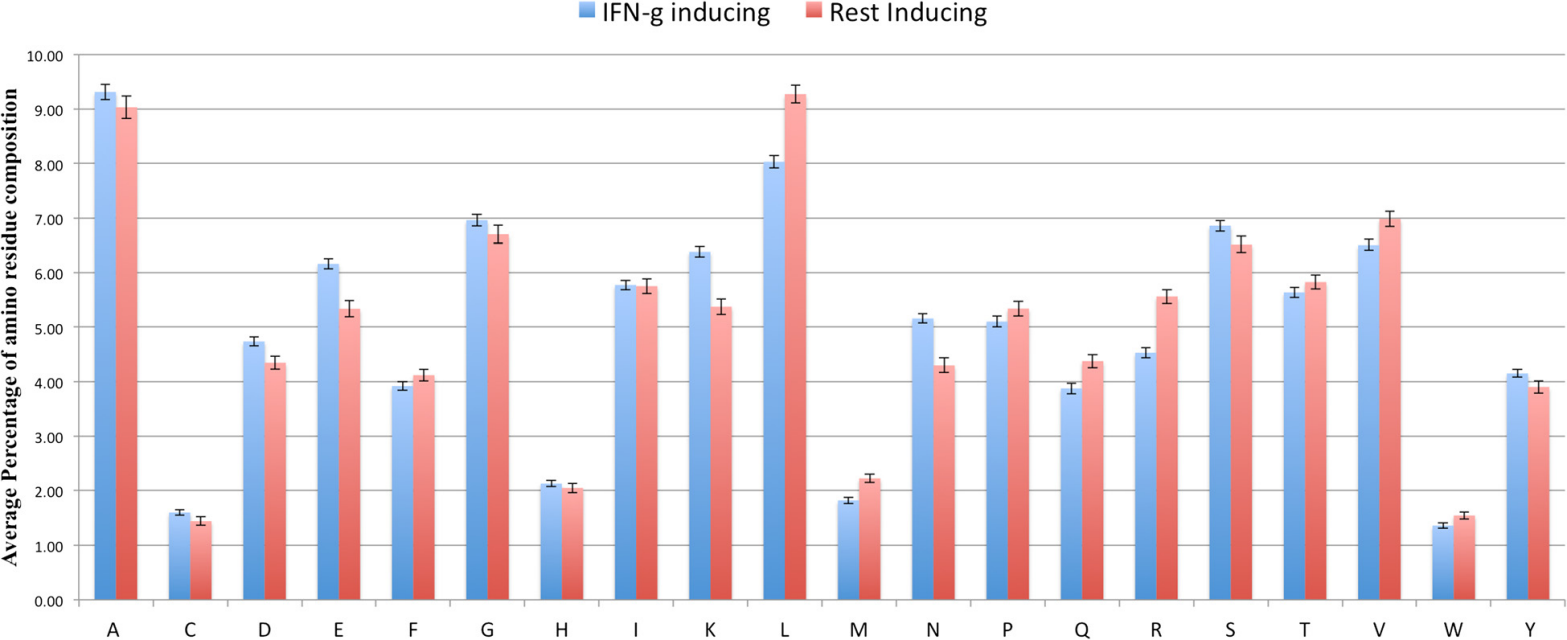
Residue composition plot for IFNgOnly dataset.

We computed amino acid composition of peptides and observed a significant difference in composition of certain residues in two types of peptides. In case of IFN-γ inducing peptides A, E, G, P, Q, R residues are more abundant, while residues C, L, S, T, I are more preferred in negative peptides (Figure 5). On the other hand the residues D, E, K and N are more abundant in IFN-γ inducing dataset as compared to the residues L, V, R and M are preferred for the induction of other cytokine than IFN-γ as depicted from two-sample logo of IFNgOnly dataset (Figure 6).

### Positional preference of residues

**Figure 7:**
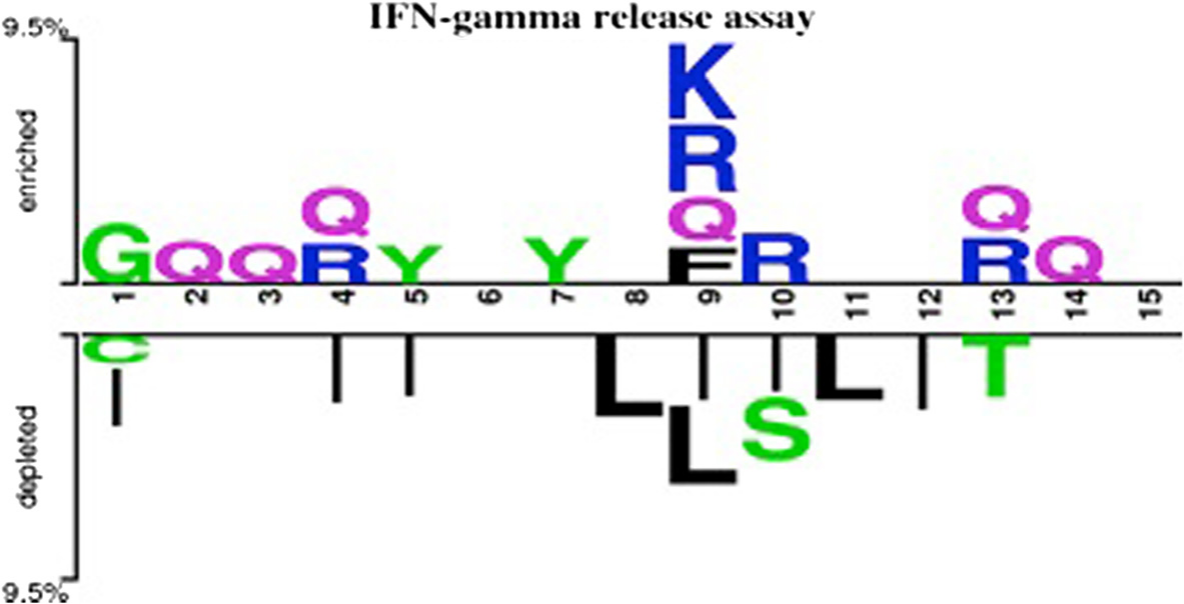
Two-sample logo of 15 N-terminal amino acids (first 15 residues) in main dataset at a p value of <0.0001.

**Figure 8:**
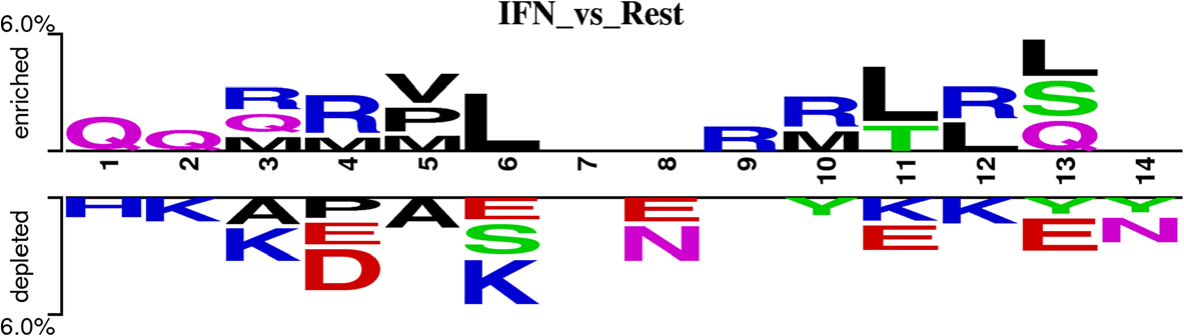
Representing the Two-sample logo from 15 N-terminal residues in IFNgOnly dataset at p value of <0.05.

Compositional analysis provides only overall preference of a residue but no information about preference of a particular residue at a specific position in peptide. In order to understand positional information of each residue; we created a two-sample logo for our positive and negative peptides. We observed that amino acids are playing an important role in discriminating the IFN-γ inducing and non-inducing peptides (Figure 7). Charged residues are preferred in positive dataset at 4^th^, 9^th^, 10^th^ and 13^th^ position, on the other hand aliphatic residues are preferred at 4^th^, 5^th^, 9^th^, 11^th^ and 12^th^ position in negative peptides. Additionally, polar uncharged residues are prevalent at 2^nd^, 3^rd^ and 14^th^ position in IFN-γ inducing instances. In case of peptides in IFNgOnly dataset, it was observed that glutamine is preferred at first to third position of IFN-γ inducing peptides while for the induction of other cytokine positively charged residues like H and R are preferred at these position (Figure 8). It is also clear from the Figure 8 that negatively charged residues are not preferred at any of position in IFN-γ inducing peptides but in case of induction of rest of cytokine negatively charged residues are prevalent at 4^th^, 6^th^, 8^th^, 11^th^ and 13^th^ position.

### Motif search

In order to discover exclusive patterns or motif in our peptides, we used the MERCI software. We have used three kinds of amino acid classification (None, Koolman-Rohm and Betts-Russell) to discover the 100 motifs. We observed that Betts-Russell classification under polar root could discriminate the dataset most significantly with 532 positive peptides and 1835 negative peptides. By combining all the motifs from different classification, 964 positive and 2827 negative peptides could be discriminated [Table 1].

**Table 1:** Exclusive motifs of different class found in IFN-γ inducing and non-inducing peptides

**Serial no.**	**Class of motifs**	**No. of exclusive pos peptide**	**No. of exclusive neg peptide**
**1**		None	71	1045
**2**		Koolman-Rohm	148	624
**3**		Betts-Russell (Hydorphobic)	501	573
**4**		Betts-Russell (Polar)	532	1835
**5**		Betts-Russell (Small)	320	1133
**6**		**ALL class**	**964**	**2827**

These motifs were discovered using MERCI software.

The top motifs from each classification are shown in Table 2. The most significant motifs discovered is “[aliphatic]-I-[aliphatic]L[aliphatic][aliphatic][aliphatic]-[aliphatic]”, which was repeated in 89 negative peptides and was absent in positive peptides. The most significant motif in positive IFN-γ inducing dataset, that is present in 53 positive sequences and none of the negative sequence, is “Q-[aliphatic]-[neutral]-P[neutral]-Q”.

**Table 2:** Frequency of best motifs discovered using MERCI software in IFN-γ epitopes and non-epitopes

**Class of Motifs**	**Found in IFN epitopes**	**Frequency**	**Found in non-epitopes**	**Frequency**
None	QPQ-Q-P-Q	41	IS-L-M	40
Koolman-Rohm	Q-[aliphatic]-[neutral]-P[neutral]-Q	53	[aliphatic]-I-[aliphatic]L[aliphatic][aliphatic][aliphatic]-[aliphatic]	66
Betts-Russell (Hydorphobic)	[aliphatic][polar][small][polar][aliphatic][polar][small]E	37	[hydrophobic]I[hydrophobic][aliphatic][aliphatic][hydrophobic][hydrophobic][hydrophobic]	89
Betts-Russell (Polar)	[polar][hydrophobic][hydrophobic][polar][small][small][aliphatic][small][small]E	32	[polar][aliphatic][hydrophobic][hydrophobic][hydrophobic][aliphatic][hydrophobic][hydrophobic][charged][hydrophobic]	40
Betts-Russell (Small)	[small][hydrophobic][polar][polar][charged]W[polar]	31	[small][aliphatic]I[aliphatic][hydrophobic][hydrophobic][small][hydrophobic]	51

Negative sign (−) = represents the gaps at that position.

MERCI software compares positive and negative dataset and motifs provided will be changed as we change the input dataset. So in case of IFNgOnly dataset the best classification is Betts-Russell under polar root for IFN-γ inducing epitopes. It was observed that 37% of IFN-γ inducing epitopes could be discriminated with this classification. While for inducing rest of cytokine (except IFN-γ) best discrimination was observed when no classification of amino acid was used, where we can predict up to 384 epitopes (Table 3).

**Table 3:** Exclusive motifs of different class found in IFN-γ inducing and other cytokine (except IFN-γ) inducing peptides from IFNgOnly dataset

**Serial no.**	**Class of Motifs**	**No. of exclusive IFN-γ peptide**	**No. of exclusive rest of cytokine inducing peptide**
**1**	None	696	384
**2**	Koolman-Rohm	1481	219
**3**	Betts-Russell (Hydorphobic)	1731	187
**4**	Betts-Russell (Polar)	1318	234
**5**	Betts-Russell (Small)	1668	186
**6**	**ALL class**	**3058**	**679**

These motifs were discovered using MERCI software.

We have also extracted the best motifs for such distinction in each classification approach for our second dataset “IFNgOnly” (Table 4) and found that “YR[aliphatic]” is the best motif in IFN-gamma inducing epitopes to discriminate them from the epitope that have induced other cytokine. This motif was present in 63 sequences. On the other hand “PN[hydrophobic][small]-[positive]-[polar]” was the most prevalent motif to distinguish epitopes that have induced other cytokine from IFN-γ inducing peptides with the coverage of 32 sequences.

**Table 4:** Frequency of best motifs discovered using MERCI software in IFN-γ epitopes and Rest of cytokine inducing epitopes from IFNgOnly dataset

**Class of Motifs**	**Found in IFN Epitopes**	**Frequency**	**Found in Rest-epitopes**	**Frequency**
None	F-QP-Q	42	ANKIR	17
Koolman-Rohm	R[basic]-R-[aliphatic] [neutral]	51	[neutral]K[aliphatic]RE	17
Betts-Russell (Hydorphobic)	YR[aliphatic]	63	[hydrophobic]-N[hydrophobic][small]K-[R]	29
Betts-Russell (Polar)	[polar]-YR[aliphatic]	53	[polar]-N[hydrophobic] [small]K-R	28
Betts-Russell (Small)	small-YR[aliphatic]	54	PN[hydrophobic][small]-[positive]-[polar]	32

Negative sign (−) = represents the gaps at that position.

### Model based on machine learning technique

In this study, we developed Support Vector Machine (SVM) based models, implemented using freely available software SVM^light^ that is widely used in classification problems [21,59-61]. In this study, we developed SVM based models using amino acid and dipeptide composition of peptides and achieved maximum MCC 0.33 and 0.49, respectively [Table 5]. It has been observed that length of peptide play vital role in discriminating these two types of peptides. Thus we also developed model using amino acid and dipeptide composition of peptides with length as an additional feature and achieved maximum MCC 0.43 and 0.54, respectively. This clearly indicates the role of length of peptides in discriminating two types of peptides.

**Table 5:** The performance of SVM based models developed using residue (amino acid) and dipeptide composition with and without length of peptides on our main dataset

**Input feature**	**Descriptors**	**Sensitivity**	**Specificity**	**Accuracy**	**MCC**
Residue Composition	20	53.2	78.8	69.71	0.33
Residue Composition + Length	21	51.82	87.77	75	0.43
Dipeptide Composition	400	62.89	84.74	76.98	0.49
Dipeptide Composition + Length	401	66.5	86.71	79.54	0.55

We have also built SVM models for IFNgOnly dataset and attained maximum MCC 0.25 and 0.35 with residue composition and dipeptide composition, respectively (Table 6). The performance of our model was not changed significantly when length was used as feature with composition.

**Table 6:** The performance of SVM based models developed using residue (amino acid) and dipeptide composition with and without length of peptides on IFNgOnly dataset

**Input feature**	**Descriptors**	**Sensitivity**	**Specificity**	**Accuracy**	**MCC**
Residue Composition	20	64.11	62.69	63.65	0.25
Residue Composition + Length	21	64.8	63.84	64.49	0.27
Dipeptide Composition	400	66.12	71.2	67.77	0.35
Dipeptide Composition + Length	401	65.31	71.9	67.45	0.35

Additionally, we also developed SVM based models using binary profile where each position is represented by a vector of dimension of 20 (each element represent presence or absence of a specific type of residue). The performance of models developed using binary profile of N-/C-terminal residues is shown in Additional file 2: Table S1 along with the composition variation plot for each residue in Additional file 2: Figures SF1 and SF2.

### Hybrid approach

**Figure 9:**
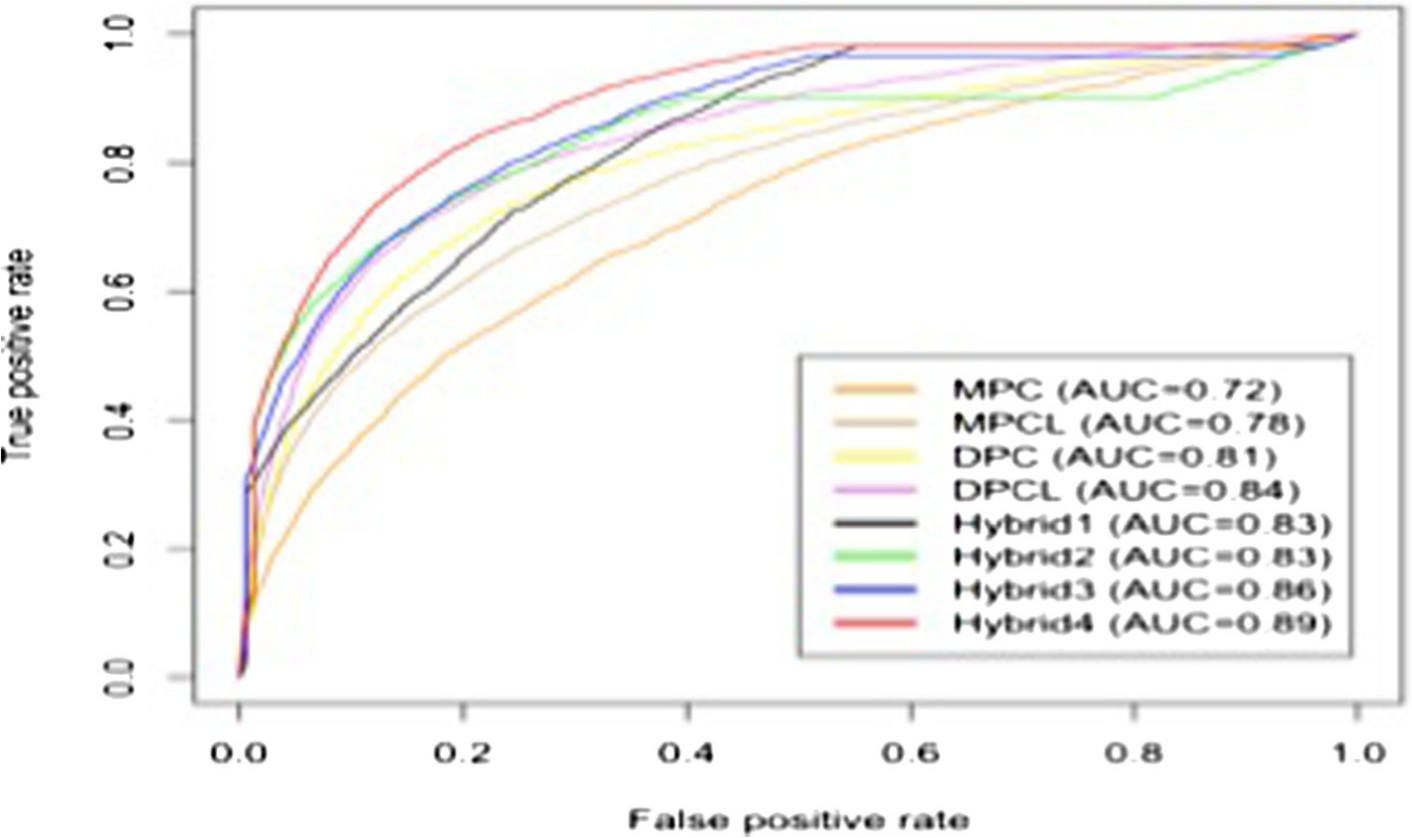
The performance of various models developed in this study in form of ROC plots on our main dataset.

The hybrid approach was applied to combine the prediction using MERCI and SVM. In this approach, the dataset were classified on the basis of exclusively motif search using MERCI, where 964 IFN-γ inducing and 2827 IFN-γ non-inducing MHC class II binders could be discriminated and the remaining 2741 positive and 3901 negative peptides were discriminated using SVM. In this approach four different hybrid models were developed with different input features. We observed that using the hybrid approach the performance was increased in each hybrid model. By this way, we achieved MCC value up to 0.62 in combining dipeptide composition, length and Merci motif search [Table 7]. The comparative results were also plotted in threshold independent manner using ROC plot (Figure 9). In order to check the robustness of model, 10 fold cross validation was performed on our best model and consistency in the performance was observed. Rest of the results were generated on 5 fold cross validation.

**Table 7:** The performance of hybrid models that combines Motif based approach with SVM models developed using residue and dipeptide composition with or without length on our main dataset

**Features**	**Sensitivity**	**Specificity**	**Accuracy**	**MCC**
Residue Composition	70.74	76.56	74.49	0.46
Residue Composition + Length	74.84	80.26	78.34	0.54
Dipeptide Composition	74.79	80.98	78.78	0.55
Dipeptide Composition + Length	**77.98**	**84.36**	**82.10**	**0.62**
Dipeptide Composition + Length (10 fold)	**78.49**	**84.39**	**82.30**	**0.62**

We have also developed the hybrid models for our second dataset (IFNgOnly). Here the MERCI could discriminate 3058 IFN-γ and 679 rest cytokine epitopes. The remaining 1425 IFN-γ and 1485 rest cytokine epitopes were discriminated using SVM. The performance of the hybrid model was 86.97%, 68.38%, 80.93% in terms of sensitivity, specificity and accuracy [Table 8]. On the other hand, performance was slightly improved with dipeptide composition feature. We also observed adding length as an additional feature in residue composition or dipeptide composition did not improve the performance [Table 8].

**Table 8:** The performance of hybrid models that combines Motif based approach with SVM models developed using residue and dipeptide composition with or without length on the IFNgOnly dataset

**Features**	**Sensitivity**	**Specificity**	**Accuracy**	**MCC**
Residue Composition	86.97	68.38	80.93	0.56
Residue Composition + Length	85.19	73.33	81.33	0.58
Dipeptide Composition	87.53	68.66	81.39	0.57
Dipeptide Composition + Length	87.31	68.89	81.32	0.57

### Models for discovering IFN-γ inducing peptides

All the models described above has been developed on dataset that contain experimentally validated IFN-γ inducing and non-inducing MHC class II binders. These models only can be used for predicting IFN-γ inducing peptides if users know that their query peptide is MHC class II binders. In order to provide service to the community we developed models on alternate dataset “IFNrandom” (random negative dataset) that can be used to discover IFN-γ peptides in proteins/antigens. As described in materials and the methods section our alternate dataset contain, negative set/examples are random peptide. We developed models on alternate dataset and achieved maximum accuracy of 73.4% and sensitivity of 69.18% (Table 9).

**Table 9:** Performance of SVM on IFNrandom dataset with compositional features of residues

**Descriptors**	**Threshold**	**Sen**	**Spec**	**Acc**	**MCC**
**AA Composition**	**0**	**64.97**	**69.04**	**67**	**0.34**
**DP Composition**	**0.1**	**69.18**	**77.62**	**73.4**	**0.47**

## Discussion

In the era of computer aided vaccine design, researchers are trying to find out the best epitope that can induce desired immune response. To be the best vaccine candidate, a peptide should not only be epitope for B and T cell, but it should also be able to evoke the desired type of immune cells to generate the required response. For example, in tuberculosis the vaccine candidate must be able to induce IFN-γ to eradicate the infection [62-64]. Therefore, there is a need of a method, which could predict the peptide responsible for secreting IFN-γ. The MHC-peptide complex may be exceptionally crucial for deciding the type of transcription factors to be activated after this association, which is responsible for the type of cytokine released [65,66]. Therefore, the biasness of MHC alleles and secretion of interferon-gamma were analyzed. In case of main dataset, alleles were not determined for 10,767 assays out of 17,752 interferon gamma assays; similarly in case of IFNgOnly dataset) alleles were not determined for 6576 assays out of 15,778. Source organisms and host species/strains are also very important in deciding the secretion of interferon gamma.

The binding affinity of these peptides with MHC was shown to be dependent on the length of peptides [67]. Therefore we analyzed the length variation in our datasets. The variability in length was observed from 9 to 30 with some exceptions, which was in consistent with previously reported by Nielsen *et. al*. in the analysis of SYFPEITHI and MHCPEP [68-70]. The skewness in the positive dataset was observed with length more than 15 amino acid residues. It has been reported that the peptides having more than 15–16 amino acids showed less affinity toward MHC class II, and this lesser affinity might be creating an environment that lead to release of IFN-gamma. We have also observed that length of the peptide is not significantly different in IFN-γ, when compared with length of peptides that have induced other cytokine.

Besides length, the conservation of the residue at a specific position may also be beneficial. Therefore, we have compared the positive and negative epitope data to fish out the prime residue activating IFN-γ releasing potential. In our observation, it was noticed that charged residues are preferred in at 4, 9, 10, 13 and 14 positions whereas, the Leucine and Iso-leucine residues are dominating in the peptides not inducing the release of IFN-γ. The differential preference may be significant for the different activation factors activated. While in case of comparison between IFN-γ and rest of cytokine using IFNgOnly dataset, it was observed that at position 4^th^, 9^th^ and 10^th^ charged residue are more prevalent in IFN-γ inducing peptides. This observation was in consensus with the observation from our main dataset. It was also found that negatively charged residues are dominating for induction of other cytokine except IFN-γ at 4^th^, 6^th^ ,8^th^ ,11^th^ and 13^th^ position. This kind of discrimination could be utilized for designing Th1 inducing peptides based upon amino acid properties.

The positional feature of a sequence could be encoded in machine learning format by generating binary feature input. This binary feature input could only be applied at a fixed length pattern, therefore different binary inputs were created by varying the length of amino acids from 9 to 15 through both N and C-terminal of a peptide. The performance of SVM model on these input vectors was nearly the same in terms of MCC. The compositional vector amino acid and dipeptide for a sequence has fixed feature input (20 and 400 respectively) irrespective of length of the peptide. The SVM performed better on these feature input as compared to binary vectors.

The performance of the SVM based models increases after adding a feature of length along with compositional vector. This may be co-related with the earlier report of variation in affinity of MHC-peptide binding with the variation in length of peptide [65]. The overhanging and short peptides may be interfering with the ternary complex of peptides-MHC-T cells. The exclusive motifs in positive or negative dataset may be a major driver for this differential behavior; these motifs were explored using MERCI software. The motifs could be searched using different classification of amino acids proposed in the literature. The best classification is Betts-Russell with hydrophobic root for our dataset. The top 100 motifs searching under hydrophobic root of Betts-Russell classification are able to cover 532 of positive peptides and 1835 of negative peptides. The significance of motifs could be estimated by its coverage and hydrophobic motifs are most commonly found in negative dataset.

## Conclusion

In past large number of methods have been developed for predicting MHC Class II binders or T-helper epitopes. In this study, an attempt has been made to classify MHC class II binders based on their interleukin induction. We classify MHC class II binders in two categories; first category of binders have ability to induce IFN-γ where as second category of binders do not have ability to induce IFN-γ. In order to discriminate two categories of MHC binders, models have been developed using various features of binders/peptide sequence that include binary pattern, compositions, and motifs. Our models were able to predict IFN-γ inducing peptide with high precision, it mean it is possible to design peptide that can induce IFN-γ. This study also indicates the preference of certain MHC alleles and host strains/species to skew the immune response to release interferon-gamma. In the near future, these prediction models will be useful in the advancement of computer aided vaccine design, where researcher will be able of designing subunit vaccine with the desired immune response.

### Webserver for designing IFN-γ inducing peptides

In order to serve scientific community, we developed a web server IFNepitope using PHP, Perl, HTML and Java scripts. This web server has three major modules called Predict, Design and Scan. Module Predict allow users to screen peptide library for predicting best IFN-γ inducing epitopes. Design module of IFNepitope allows to identify minimum mutations required in a peptide to make it IFN-γ inducing epitope. In Design module, first all possible single residue mutation peptides are generated then module predict IFN-γ inducing epitope in mutant peptides. Similarly, Scan module predict antigenic or IFN-γ inducing regions in an antigen. Overall this server will be useful for researchers working in the field of subunit vaccines.

## Reviewers’ comments

**Reviewer number1:** Prof Kurt Blaser

**Comment:** The paper is interesting and helpful for prediction and modulation of antigenic compounds and generation of Type I cytokine pattern mainly in protective immunizations but also for allergen-immunotherapy. The approach, although it is based on published experimental observations, on theoretical mathematical models. Thus it is difficult for me to evaluate the value and correctness of these predictions. What to my mind is missing, are some experimental data from human in vitro experiments, either with specific T cell clones or adequate PBMC cultures that are stimulated with synthetic epitope- peptides from these models. It is furthermore important that in such experiments not only IFN-gamma but also a broader pattern of the most important cytokines are measured, as in many cases rather the ratio of IFN-gamma: other cytokines (e.g. IL4) is important and not the absolute amount of IFN-gamma.

Thus, I would strongly recommend to add such experimental data in order to prove the effectiveness of the described models.

**Response:** We understand the reviewer’s concern about the experimental validation of our model, but also its noteworthy to mention here that we have developed this model on experimentally proven dataset and evaluated using well-established computational cross-validation approaches.

Quality of written English: Needs some language corrections before being published.

**Reviewer number2:** Prof Laurence Eisenlohr

In this manuscript, Dhanda et al. describe their efforts to develop tools to predict those MHC class II-binding peptides that induce interferon-gamma production and those that do not.

My concerns with this paper are as follows:

**Comment:** 1) The authors mined the peptide sequences they analyzed from the Immune Epitope Database. My understanding (from communicating with IEDB staff) is that those peptide sequences listed as “Negative” for cytokine production have not been shown to bind any particular MHC class II molecule (they are not “epitopes” per se, just sequences that failed to elicit a T cell response with the MHC class II restrictions that were tested). Thus, there may not be much basis for comparison; if they don’t bind MHC class II to begin with, then of course they won’t elicit interferon-gamma.

**Response:** We are thankful to the reviewer for this nice comment. To answer this comment, we have created another dataset “IFNgOnly”, which comprises the peptides, which induce any other cytokine except interferon-gamma. We have applied the same approach and achieved the convincing results (Tables 3, 4, 6, 8 and 9).

**Comment:** 2) I imagine that most of these negative sequences are derived from overlapping15-16-mer peptide libraries that were comprehensively screened for immunogenicity. This seems to be the likely explanation for the skewing of negative sequences toward that size range.

**Response:** We do agree with the reviewer and therefore produced results with alternative negative dataset, and observed no peptide length-wise preference for the induction of interferon-gamma when compared with the peptides that have induced other cytokine (except IFN-gamma) in IFNgOnly dataset as shown in Figure 4.

**Comment:** 3) There is no consideration of species origin of the class II molecule or MHC polymorphism. A negative peptide sequence for one animal or MHC allele could be strongly positive in other conditions if they were to be tested.

**Response:** This is important issue raised by reviewer, we examine our main dataset again after comment. We analyzed the IFN-gamma response with respect to MHC alleles and found that there are 38 peptides in our dataset that elicit IFN-gamma response in one host/MHC allele and did not elicit such response with another host/MHC-allele. We have considered these epitopes in our positive examples.

**Comment:** 4) As far as I can tell, there is also no consideration of peptide binding register (where the peptide is positioned with respect to the binding pockets). Therefore, I do not know what to make of the reported positional effects.

**Response:** We agree with the reviewer that positional preference analyzed by us could not be correlated with MHC groove because the positional information of peptides. It is also fact that for most of the peptides/binders position in MHC binding grove is not known. This is first study and in future these points should be addressed when sufficient data is available.

Quality of written English: Needs some language corrections before being published

**Response:** We have revised the manuscript and corrections have been made to improve the English.

**Reviewer number3:** Dr Manabu Sugai

The authors have an idea to find ideal peptides to elicit Th1 response for developing novel vaccination strategy. To this end, they developed a webserver for predicting IFN-gamma inducing peptides by analyzing the dataset from IEDB.

**Comment:** The paper is interesting, and I think would be of interest to readers of Biology Direct. However, the validation of their program is not enough for providing functional rationale to support their concept. Author’s idea depends on the notion that the specific peptides promote specific helper T cell differentiation. However, such an idea is not easily accepted, because various cytokines, but not TCR-signals, play dominant role in instructing helper T cell differentiation.

**Response:** Thank you for this comment, but we would like to draw your intention to some of references, where substitution of single amino acid in a peptide had skewed the immune response from Th1 to Th2 or vice-versa [49-52]. Therefore the notion of peptide based immune modulation is existing in literature and we are developing a prediction model for designing the peptide that have potential to induce IFN gamma and hope that this model would be very useful in peptide-based vaccination and therapeutics.

**Comment:** On the other hands, IFN-gamma inducing activities of the peptides were usually estimated as a memory reaction, indicated by the comments from IEDB. Therefore, we can speculate that some peptides specific immune reaction occurs specifically in Th1 skewed condition. According to this notion, we can use IFN-gamma inducing peptides as an adjuvant to induce memory reaction in vivo.

To validate this notion, the authors need to examine whether other cytokine-inducing-peptides, such as IL4, IL17, TGF-b etc., are selected or not by IFN-gamma inducing-peptides finding program. If your program excludes other cytokine-inducing-peptides, your ideas are supported partially and provide the meaning of your program for future use. If other cytokine-inducing-peptides are also included in the selected IFN-gamma inducing-peptides, author’s concept is not correct or program itself is incomplete.

**Response:** We are thankful to reviewers for providing detail information on IFN-gamma inducing peptides. Our aim in this study is to discriminate inducing and non-inducing peptides. In order to address issue raised by reviewer we developed models for discriminating IFN-gamma and non-IFN-gamma (induce other cytokines except IFN-gamma). First we created a dataset called IFNgOnly contains IFN-gamma and other cytokine inducing peptides. The performance of our models on this IFNgOnly dataset is shown in Tables 3, 4, 6 and 8. As shown in result section our models were able to discriminate peptide which induce IFN-gamma and peptides that induce other cytokines.

Quality of written English: Acceptable

Responses to reviewer’s comments after revision

Reviewer number:2 Prof Laurence Eisenlohr

This paper by Dhanda and et al. proposes both length and sequence biases for MHC class II presented peptides that elicit interferon-gamma responses (vs. those that do not). I remain skeptical about the conclusions in this paper due to lack of information on:

**Comment: 1)** the basis for lack of interferon production. Are peptides that have been shown to bind to an MHC class II molecule but not elicit any response (“non-epitopes”) included in the analyses? This would be problematic. In our hands essentially all epitopes elicit some interferon-gamma response following natural virus infection so the distribution of epitopes (3705 inducers vs. 6728 non-inducers) is worrisome.

**Response:** Our main dataset contain 10,433 peptides obtained from 17,752 IFN-gamma assays; 6,728 peptides do not release IFN-gamma (as per IEDB) which we called negative peptides in this study. We agree with reviewer that all the MHC class II binders are not epitopes; thus negative peptides in our main dataset may also include non-epitope. In order to overcome this limitation, we created another dataset called IFNgOnly, where negative peptides/epitopes contain only those peptide that induce cytokine other than IFN-gamma. In simple term negative peptides in IFNgOnly are true non IFN-gamma inducing epitopes.

**Comment: 2)** inclusion/exclusion criteria. As an exercise, I went to the IEDB and searched for all MHC class II binders that do not elicit an interferon-gamma response. I then randomly chose lysteriolysin O (LLO), residues 216-227, described by Skoberne et al., 2002, J Immunol., 169:1410-8. In fact, this epitope does elicit an interferon-gamma response in BALB/c mice (because it is a CD4+ T cell epitope in that strain) but does not elicit an interferon-gamma response in C57Bl/6 mice (because it is not an epitope in that strain). Thus, it is listed in both categories. How did the authors deal with this? Exclude? Include in both categories? Include in only one category? How many other peptides in the database also fall into both categories?

**Response:** We followed IEDB recommendations, in case multiple assays are performed to test a peptide, it is considered positive even if a single assays shows positive. There are 667 epitopes/peptides falling in both the categories and we have included them in our positive dataset.

**Comment: 3)** the peptide lengths that are entered into the database.

Many epitopes are now identified via overlapping 15-mer libraries, with no subsequent attempts to map the minimal epitope or the effects of flanking residues due to the added expense. This seems to be the likely reason for the predominance of peptides of that size (Figure 2). In fact, the analysis can only be done with peptides that have been stringently defined with respect to the minimal core and flanking sequence effects, a much smaller set than was analyzed.

Many of the longer peptides may not have been identified by the library method but by the previous method of enzymatic digestion of antigen and in vitro assay with a T cell line, clone or hybridoma, again with no subsequent mapping of core and flanking sequences.

Also, the longer peptides may have been deduced in mapping a known response, and this could be the reason for bias toward interferon-gamma production in this cohort.o There is no discussion of the bimodal length distribution, which, for the reasons discussed, may have a technical vs. biological basis.

**Response:** Our main dataset were created without considering the epitopic information’s. Most of the peptides in our datasets are either ‘exact epitopes’ or ‘epitope containing region’ as per mentioned in IEDB database.

**Comment: 4)** how several other potential biasing factors, all of which can strongly influence both parameters (length and sequence bias) were accounted for, including:

origin of the peptide (pathogen, self-protein, natural sequence or variant, …)

host species

method of immunization (peptide, organism-experimental infection, organism-natural infection, adjuvant, …)

host strain (BALB/c vs. C57Bl/6-Type I vs. Type 2)

identity of the class II molecule. Some class II molecules are over-represented in the database and this alone could account for the deduced sequence preferences.

**Response:** We do agree with the reviewer that the issues related to host species, immunization protocol and MHC alleles should also be considered, but this is the first study to predict the immune response of a peptide sequence. These limitations have to be addressed in future research. In order to address this issue we have investigated our dataset and provided the insight in the ‘Examination of dataset’ paragraph of ‘Result’ section.

**Quality of written English:** Needs some language corrections before being published. **Reviewer number:3 Dr Manabu Sugai**

The revised manuscript from Dhanda et al. has been significantly improved. This paper is acceptable now.

**Quality of written English:** Acceptable

## Authors’ contributions

SKD developed prediction models and webserver under the supervision of GPSR. Manuscript was written by SKD, PV and thoroughly revised by GPSR. All authors read and approved the final manuscript. All authors read and approved the final manuscript.

## Competing interest

The authors declare that they have no competing interests.

## Supplementary Material

Additional file 1 **Analysis of epitopes with respect to MHC allele, host strain and source organism.** There is 6 different sheets in this excel file: (1) The distribution of peptides with respect to (w.r.t.) source organisms in our main dataset. (2). The distribution of peptides w.r.t. host strains in our main dataset. (3) The distribution of peptides w.r.t. MHC alleles in our main dataset. (4) The distribution of peptides w.r.t. source organism in our IFNgOnly dataset. (5) The distribution of peptides w.r.t. host strains in our IFNgOnly dataset. (6) The distribution of peptides w.r.t. MHC alleles in our IFNgOnly dataset. Click here for file

Additional file 2 **Performance of SVM light on 15 residues from N or C terminal. Table S1.** The performance of various features with SVM light on 5 folds cross validation. **Figure SF1.** Variation in amino acid residue composition of residue taken from 15 N-terminus. **Figure SF2.** Variation in amino acid residue composition of residue taken from 15 C-terminus. Click here for file
