# Supplementary material for: Designing of interferon-gamma inducing MHC class-II binders
Source: Biol Direct. 2013 Dec 5;8:30. doi: 10.1186/1745-6150-8-30 (PMC4235049; doi:10.1186/1745-6150-8-30)

Supplementary Information

Table S1: The performance of various features with SVM light on 5 folds cross validation

Features	No.	Pos*	Neg*	Thr	Sen	Spec	Acc	MCC	AUC
**Binary -9aa Nterminal**	180	3691	6719	0.1	44.05	78.61	66.36	0.24	0.67
**Binary -10aa Nterminal**	200	3545	6656	0.1	49.22	75.23	66.19	0.25	0.68
**Binary -11aa Nterminal**	220	3485	6553	-0.1	40.55	82.39	67.86	0.25	0.68
**Binary -12aa Nterminal**	240	3430	6549	-0.2	64.75	61.05	62.32	0.25	0.68
**Binary -13aa Nterminal**	260	3219	6461	0.1	30.38	90.53	70.53	0.27	0.69
**Binary -14aa Nterminal**	280	3108	6392	0.1	27.19	91.91	70.74	0.26	0.68
**Binary -15aa Nterminal**	300	2965	6336	-0.3	58.04	68.83	65.39	0.26	0.69
**AAC -9aa Nterminal**	20	3691	6719	0.2	24.28	93.02	68.65	0.25	0.66
**AAC -10aa Nterminal**	20	3545	6656	-0.3	42.99	81.81	68.32	0.27	0.68
**AAC -11aa Nterminal**	20	3485	6553	-0.1	24.94	94.25	70.18	0.28	0.67
**AAC -12aa Nterminal**	20	3430	6549	-0.1	26.56	93.45	70.46	0.28	0.67
**AAC -13aa Nterminal**	20	3219	6461	0	22.34	95.08	70.89	0.27	0.68
**AAC -14aa Nterminal**	20	3108	6392	-0.3	41.83	84.28	70.39	0.29	0.69
**AAC -15aa Nterminal**	20	2965	6336	-0.2	35.35	89.68	72.36	0.3	0.7
**DPC -9aa Nterminal**	400	3691	6719	0.1	38.31	89.3	71.22	0.33	0.72
**DPC -10aa Nterminal**	400	3545	6656	0	47.22	84.95	71.84	0.35	0.73
**DPC -11aa Nterminal**	400	3485	6553	0.1	49.01	86.43	73.44	0.39	0.75
**DPC -12aa Nterminal**	400	3430	6549	-0.3	53.99	84.07	73.73	0.4	0.76
**DPC -13aa Nterminal**	400	3219	6461	-0.5	76.51	67.82	70.71	0.42	0.76
**DPC -14aa Nterminal**	400	3108	6392	-0.5	74.29	72.65	73.19	0.45	0.77
**DPC -15aa Nterminal**	400	2965	6336	-0.5	72.18	75.77	74.63	0.46	0.78
**AAC -9aa Cterminal**	20	3691	6719	-0.2	32.16	87.53	67.9	0.24	0.66
**AAC -10aa Cterminal**	20	3545	6656	-0.3	18.98	96.72	69.71	0.26	0.67
**AAC -11aa Cterminal**	20	3485	6553	-0.3	23.33	94.26	69.64	0.26	0.67

**AAC -12aa Cterminal**	20	3430	6549	-0.2	52.22	74.45	66.81	0.27	0.68
**AAC -13aa Cterminal**	20	3219	6461	-0.3	50.45	76.89	68.1	0.28	0.69
**AAC -14aa Cterminal**	20	3108	6392	-0.3	40.6	84.18	69.93	0.27	0.68
**AAC -15aa Cterminal**	20	2965	6336	0	36.49	87.96	71.55	0.29	0.69
**Binary -9aa Cterminal**	180	3691	6719	-0.2	20.78	95.88	69.25	0.27	0.67
**Binary -10aa Cterminal**	200	3545	6656	-0.2	17	98.46	70.15	0.29	0.68
**Binary -11aa Cterminal**	220	3485	6553	-0.3	17.73	97.82	70.01	0.28	0.68
**Binary -12aa Cterminal**	240	3430	6549	-0.3	16.64	98.2	70.03	0.27	0.68
**Binary -13aa Cterminal**	260	3219	6461	-0.5	15.63	98.51	70.95	0.28	0.66
**Binary -14aa Cterminal**	280	3108	6392	-0.2	12.39	98.9	70.6	0.25	0.66
**Binary -15aa Cterminal**	300	2965	6336	-0.1	44.35	79.34	68.19	0.25	0.68
**DPC -9aa Cterminal**	400	3691	6719	-0.1	50.28	80.1	69.53	0.32	0.71
**DPC -10aa Cterminal**	400	3545	6656	-0.1	56.59	79.54	71.56	0.37	0.74
**DPC -11aa Cterminal**	400	3485	6553	-0.2	60.86	76.19	70.87	0.37	0.74
**DPC -12aa Cterminal**	400	3430	6549	0	51.31	85.75	73.92	0.4	0.76
**DPC -13aa Cterminal**	400	3219	6461	-0.2	67.26	73.89	71.68	0.4	0.76
**DPC -14aa Cterminal**	400	3108	6392	-0.2	59.68	81.71	74.51	0.42	0.77
**DPC -15aa Cterminal**	400	2965	6336	-0.3	59.33	83.63	75.88	0.44	0.78

*** Variation in the number of positive and negative dataset on window length is because we have considered only those epitopes that have length above or equal to that particular window length.**

Figure SF1: Variation in amino acid residue composition of residue taken from 15 N-terminus

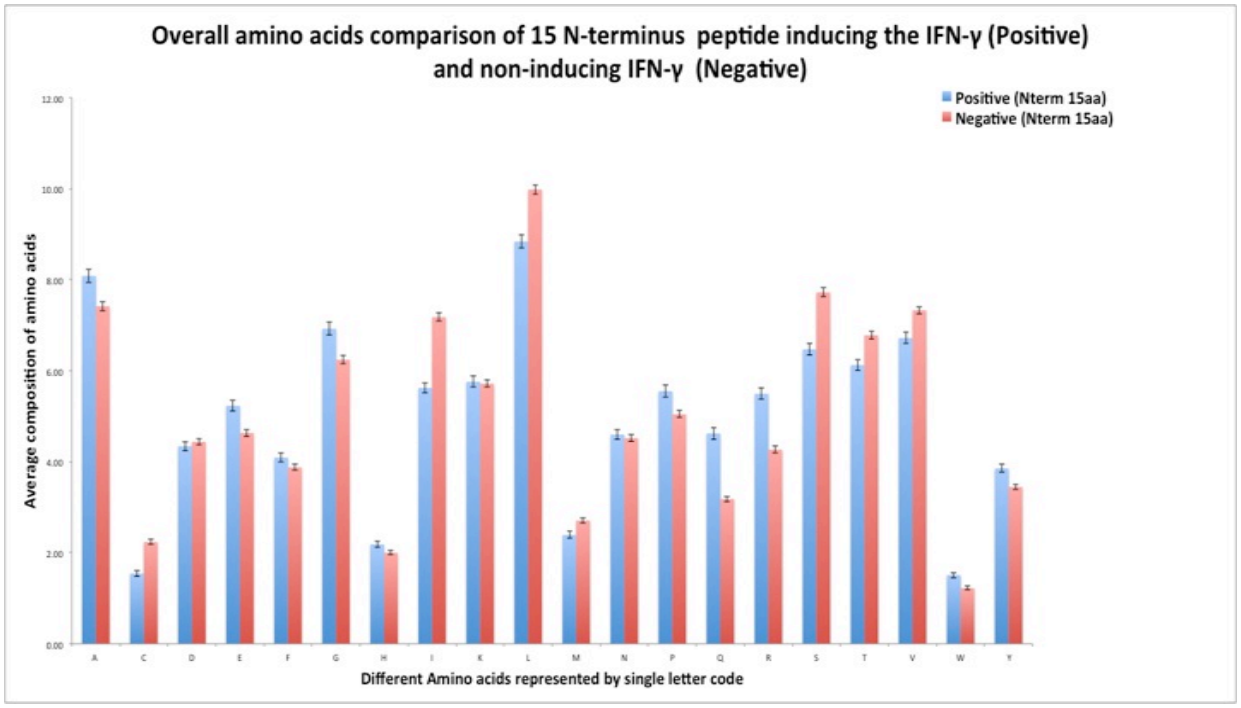

Figure SF2: Variation in amino acid residue composition of residue taken from 15 C-terminus

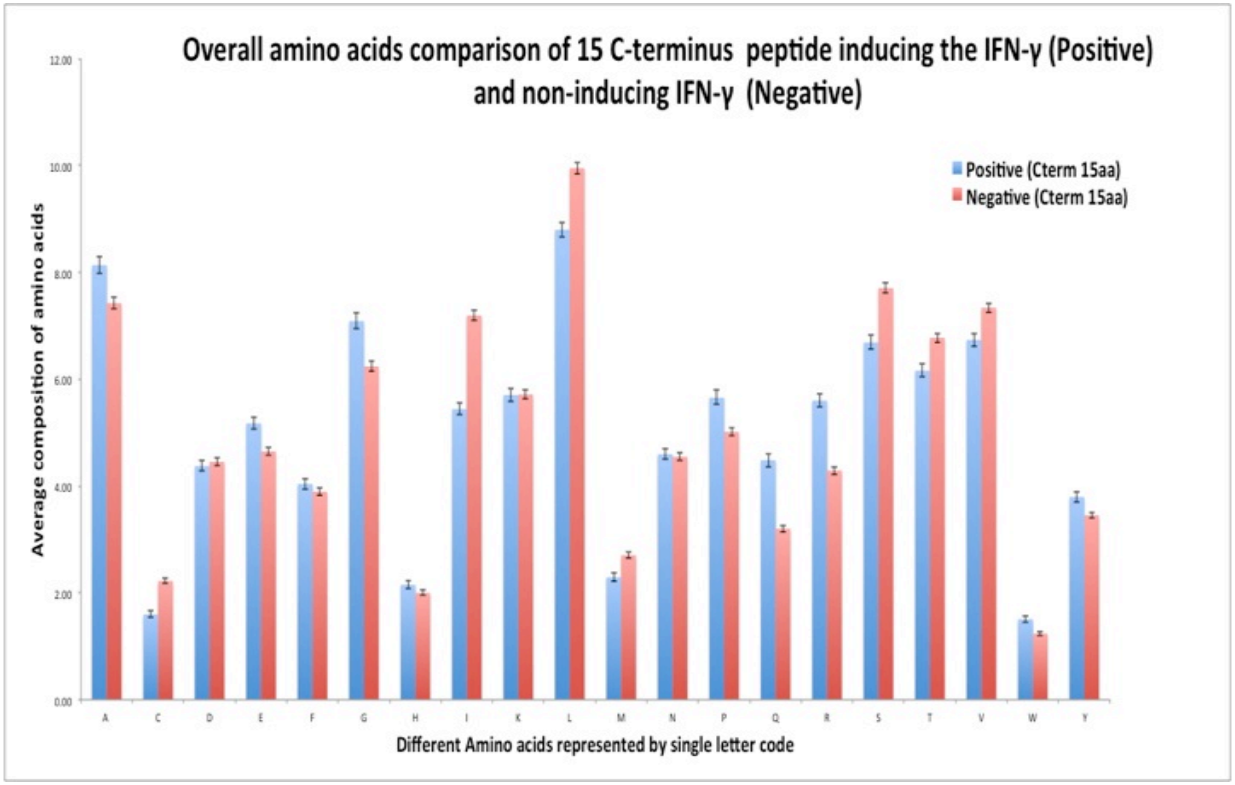

Supplement: Additional file 2 — Performance of SVM light on 15 residues from N or C terminal. Table S1. The performance of various features with SVM light on 5 folds cross validation. Figure SF1. Variation in amino acid residue composition of residue taken from 15 N-terminus. Figure SF2. Variation in amino acid residue composition of residue taken from 15 C-terminus. [file 1745-6150-8-30-S2.pdf]
